# Assessment of Factors Associated With Parental Perceptions of Voluntary Decisions About Child Participation in Leukemia Clinical Trials

**DOI:** 10.1001/jamanetworkopen.2021.9038

**Published:** 2021-05-04

**Authors:** Paula Aristizabal, Arissa K. Ma, Nikhil V. Kumar, Bianca P. Perdomo, Courtney D. Thornburg, Maria Elena Martinez, Jesse Nodora

**Affiliations:** 1 Division of Pediatric Hematology/Oncology, Department of Pediatrics, University of California San Diego, La Jolla; 2 Peckham Center for Cancer and Blood Disorders, Rady Children’s Hospital San Diego, San Diego; 3 Population Sciences, Disparities and Community Engagement, University of California San Diego Moores Cancer Center, La Jolla; 4 School of Medicine, University of California San Diego, La Jolla; 5 Now with MemorialCare Health System, Fountain Valley, California; 6 Department of Family Medicine and Public Health, University of California, San Diego, La Jolla

## Abstract

**Importance:**

Obtaining voluntary informed consent prior to enrollment in clinical trials is a fundamental ethical requirement.

**Objective:**

To assess whether health literacy, contextual factors, or sociodemographic characteristics are associated with perception of voluntariness among parents who had consented for their child’s participation in a leukemia therapeutic clinical trial.

**Design, Setting, and Participants:**

This cross-sectional study prospectively enrolled 97 parents of children diagnosed as having leukemia at Rady Children’s Hospital San Diego, a large tertiary academic center in California, from 2014 to 2017. Health literacy, contextual factors (acculturation, decisional regret, and satisfaction with informed consent), sociodemographic characteristics, and perception of voluntariness after consenting for a therapeutic clinical trial were measured. Univariable and multivariable regression were used to determine significant associations. The analyses for the present study were conducted from May 2019 to May 2020.

**Exposures:**

Informed consent for a therapeutic leukemia clinical trial.

**Main Outcomes and Measures:**

The primary outcome of interest was perception of voluntariness and its associations with health literacy and other contextual factors (acculturation, decisional regret, and satisfaction with informed consent) and sociodemographic characteristics, including age, race/ethnicity, parental language, educational level, insurance type, marital status, and socioeconomic status.

**Results:**

Of 97 parents included, the majority were women (65 [67%]), married (71 [73%]), and of self-reported Hispanic ethnicity (50 [52%]). Lower perception of voluntariness was significantly associated with lower health literacy (*r* *=* 0.30; 95% CI, 0.11-0.47; *P* = .004), Spanish language (*x̅* = −4.50, *P* = .05), lower acculturation if of Hispanic ethnicity (*r* = 0.30; 95% CI, 0.02-0.54; *P* = .05), greater decisional regret (*r* = −0.54; 95% CI, −0.67 to −0.38; *P* < .001), and lower satisfaction with informed consent (*r* = 0.39; 95% CI, 0.21-0.54; *P* < .001) in univariable analysis. Lower health literacy remained significantly associated with lower perception of voluntariness in multivariable analysis after adjustment for contextual factors and sociodemographic characteristics (β = 4.06; 95% CI, 1.60-6.53; *P* = .001). Lower health literacy was significantly associated with Hispanic ethnicity (mean, 4.16; 95% CI, 3.75-4.57; *P* < .001), Spanish language spoken at home (mean, 3.17; 95% CI, 1.94-4.40; *P* < .001), high school or less educational level (mean, 3.41; 95% CI, 2.83-3.99; *P* < .001), public insurance (mean, 4.00; 95% CI, 3.55-4.45; *P* < .001), and unmarried status (mean, 3.71; 95% CI, 2.91-4.51; *P* = .03).

**Conclusions and Relevance:**

Among parents of children with newly diagnosed leukemia who had consented for their child’s participation in a therapeutic clinical trial, lower perception of voluntariness was significantly associated with lower health literacy. These results suggest that parents with low health literacy may perceive external influences in their decision for their child’s participation in clinical trials. This finding highlights the potential role of recruitment interventions tailored to the participant’s health literacy level to improve voluntary informed consent in underserved populations.

## Key Points

**Question:**

Are health literacy, contextual factors, or sociodemographic characteristics associated with parental perception of voluntariness during informed consent for pediatric leukemia clinical trials?

**Findings:**

In this cross-sectional study that included 97 parents of children with newly diagnosed leukemia, lower perception of voluntariness was significantly associated with lower health literacy.

**Meaning:**

Low health literacy may have a role in parents not making complete and meaningful informed decisions for their child’s participation in cancer clinical trials.

## Teaser

This cross-sectional study assesses whether health literacy, contextual factors, or sociodemographic characteristics are associated with perception of voluntariness among parents who consent for their child’s participation in a leukemia therapeutic clinical trial.

## Introduction

Leukemia is the most common childhood cancer in the US,^1^ and survival rates have improved markedly in part owing to the successful recruitment of children into clinical trials.^2,3,4^ It is an ethical imperative that parents and legal guardians fully understand their role in decision-making when giving permission for their children to participate in research.^5^

Informed consent can be termed *complete and meaningful* if competence, information disclosure, comprehension, and voluntariness are effectively satisfied.^6,7^ The process involves the consenting clinician verifying the participant’s understanding of risks, benefits, and alternatives and ensuring patient’s decision-making abilities.^3,5^ Voluntariness is defined as the willingness to participate in research without feeling pressured or influenced.^8^ Recruitment into pediatric cancer clinical trials often occurs under tremendous emotional stress due to the potentially fatal nature of cancer and the need to start treatment urgently, which may negatively influence voluntariness for participation in the clinical trial.^9^

Health literacy (HL) is defined as the degree to which individuals are able to process health information to make appropriate health decisions.^10^ In the US, at least 1 in 4 adults have limited HL skills, and low HL is associated with racial/ethnic minority status and poor health outcomes.^11,12^ There is little research investigating the association of HL and other contextual factors and sociodemographic characteristics with the perception of voluntariness during informed consent for pediatric leukemia clinical trials, which comprise more than 50% of all pediatric cancer clinical trials.

To address these gaps, our study assessed the association of HL, other contextual factors (acculturation, decisional regret, and satisfaction with informed consent), and sociodemographic characteristics with the perception of voluntariness among parents of children with newly diagnosed leukemia who consented to have their child participate in a therapeutic clinical trial at our institution. We hypothesized that parents with low HL have lower perception of voluntariness compared with parents with high HL.

## Methods

### Study Participants

Our cross-sectional study included parents or legal guardians of children with newly diagnosed leukemia receiving treatment at Rady Children’s Hospital San Diego (RCHSD), a large tertiary pediatric cancer center in California. This study followed the Strengthening the Reporting of Observational Studies in Epidemiology (STROBE) reporting guideline for cross-sectional studies. The institutional review board for the University of California San Diego and RCHSD approved this study. Written consent was obtained in a manner consistent with the Common Rule requirements from participants meeting the eligibility criteria and who voluntarily chose to participate. No one received compensation or was offered any incentive for participating in this study.

Children aged 0 to 17 years who were diagnosed as having leukemia between October 2014 and June 2017 were identified from RCHSD’s Cancer Registry. Parents of these children were eligible to participate if (1) their child was eligible for a therapeutic clinical trial; (2) they had consented to participate in the therapeutic clinical trial within the previous 7 days; (3) they were a primary decision-maker; and (4) they had an understanding of written and spoken English or Spanish. We excluded parents who had given consent for any therapeutic clinical trial in the past, had a child with a second malignant neoplasm or relapsed disease, had a child previously diagnosed as having leukemia at an outside institution, or would potentially transfer cancer care to another institution.

### Study Procedures

Our primary outcome of interest was voluntariness and its associations with HL and other contextual factors (acculturation, decisional regret, and satisfaction with informed consent) and sociodemographic characteristics. Participants completed questionnaires within a week of the informed consent discussion to assess perception of voluntariness, HL, acculturation (if Hispanic), decisional regret, satisfaction with informed consent, and sociodemographic characteristics (ie, age, sex, race/ethnicity, primary language spoken at home, educational level, marital status, and occupation). Questionnaires were available in both English and Spanish and administered by bilingual (English and Spanish) and bicultural (Anglo and Hispanic) research staff.^13,14,15,16,17,18^ All survey questions were written with a low level of complexity and high readability^19^ and did not contain health information that would require high HL skills to understand.

We collected data from the medical record for each patient including age, sex, and insurance type. Socioeconomic status was calculated using the Hollingshead index.^13^ Race/ethnicity was self-reported by parents, and we used US Census Bureau categories.^20^

### Study Measures

We assessed HL using the Newest Vital Sign (NVS), a 6-item test that evaluates interpretation of information from a nutrition label, with an emphasis on using numeracy skills.^21^ The NVS scores range from 0 to 6 and (with lower scores indicating limited HL), and the NVS has been validated in other disciplines^22^ and used widely, including for parents who make health decisions for their children.^23,24,25^ Perception of voluntariness was assessed using the Decision-making Control Instrument, a 9-item questionnaire with a Likert scale response format to statements such as “I was powerless in the face of this decision.”^15^ Decision-making Control Instrument scores range from 9 to 54 and (with lower scores indicating lower perception of voluntariness) and has been validated for parents who make decisions regarding research therapeutic protocols for serious childhood illnesses, including cancer.^26^ Decisional regret was assessed using the Decision Regret Scale, a 5-item questionnaire with a Likert scale,^16^ with scores ranging from 0 to 100 (lower scores indicating lower regret), and has been used for parents of children with cancer.^27^ The scale has been validated in both adult oncology patients and their caregivers.^28^ Acculturation was assessed using the Short Acculturation Scale for Hispanics, which evaluates factors of language use, media, and social relations among Hispanic individuals,^18^ with scores ranging from 12 to 60 (lower scores indicating lower levels of acculturation). The NVS and the Short Acculturation Scale for Hispanics were validated in English and Spanish.^18^ For instruments that have not been validated in Spanish, including the Decision-making Control Instrument, Decision Regret Scale, satisfaction and demographics questionnaires, we used the Brislin method, which has been extensively used in cross-cultural research.^29^

### Statistical Analysis

The primary outcome of perception of voluntariness was analyzed in association with sociodemographic characteristics using the Kruskal-Wallis and Mann-Whitney tests for robust analysis of group differences for each outcome. Spearman correlation analysis was used to determine significant associations between perception of voluntariness, HL, decisional regret, satisfaction with informed consent, and acculturation. A multivariable generalized estimating equation model was fit with perception of voluntariness as the outcome variable and HL, language, and acculturation level as significant risk factors (*P* *<* .10 from univariable models).

We analyzed HL and associated sociodemographic characteristics among study participants and conducted secondary analyses for decisional regret and satisfaction with informed consent to understand associations in related contextual factors. Significant associations were defined as *P* ≤ .05. All analyses were conducted from May 2019 to May 2020 using statistical software program R, version 3.5.0 (R Project for Statistical Computing).^30^

## Results

### Participant Characteristics

In total, 177 patients aged 0 to 17 years were diagnosed as having leukemia during the study period, and 135 parents met inclusion criteria and were prospectively approached. Of these, 26 eligible parents (19%) declined, and 12 enrolled participants (9%) withdrew from the study within 3 months of enrollment. Reasons for declining or withdrawing included feeling overwhelmed and having no time to complete questionnaires. The final sample included 97 participants, consisting of 50 Hispanic parents (52%), and 47 non-Hispanic parents (48%). The majority of participants were women (65 [67%]), married (71 [73%]), and spoke primarily English at home (51 [53%]). Sociodemographic characteristics are given in Table 1.

**Table 1.** Baseline Characteristics of 97 Study Participants (Parents or Guardians) at Rady Children’s Hospital San Diego

Characteristic	Participants, No. (%)
Age range, y	
18-34	39 (40)
35-44	44 (45)
45-75	14 (15)
Sex	
Female	65 (67)
Male	32 (33)
Race	
White	67 (69)
Mixed or multiple	13 (14)
Asian, PI, AI, or AN	12 (12)
Black	5 (5)
Ethnicity	
Hispanic	50 (52)
Non-Hispanic	47 (48)
Language spoken at home	
English	51 (53)
Spanish	39 (40)
Other	7 (7)
Level of education	
High school or less	20 (21)
Some college or technical school	42 (43)
Bachelor’s degree	22 (23)
Professional degree	13 (13)
Insurance type	
Private	49 (51)
Medicaid	48 (49)
Marital status	
Married	71 (73)
Unmarried^a^	26 (27)

Abbreviations: AI, American Indian; AN, Alaska Native; PI, Pacific Islander.

^a^ Unmarried includes single, divorced, widowed, and separated.

### Perception of Voluntariness Associated With Contextual Factors and Sociodemographic Characteristics

In univariable analysis, lower perception of voluntariness was significantly associated with lower HL (*r* *=* 0.30; 95% CI, 0.11-0.47; *P* = .004), lower acculturation if Hispanic ethnicity (*r* = 0.30; 95% CI, 0.02-0.54; *P* = .05), greater decisional regret (*r* = −0.54; 95% CI, −0.67 to −0.38; *P* < .001), and lower satisfaction with informed consent (*r* = 0.39; 95% CI, 0.21-0.54; *P* < .001) (Table 2). Lower perception of voluntariness was significantly associated with Spanish as the primary language spoken at home (*x̅* = −4.50; *P* = .05). Parental age, race, Hispanic ethnicity, educational level, insurance type, marital status, and socioeconomic status were not associated with perception of voluntariness (Table 2). In multivariable analysis, after adjustment for contextual factors and sociodemographic characteristics of primary language spoken at home, acculturation level, satisfaction with informed consent, and decisional regret, HL remained significantly associated with parental perception of voluntariness (β = 4.06; 95% CI, 1.60-6.53; *P* = .001) (Table 3).

**Table 2.** Univariable Analysis of Perception of Voluntariness Associated With Sociodemographic Characteristics and Contextual Factors

Variable	Mean (SD) [95% CI]
Age range, y	
18-34	45.46 (7.27) [43.04 to 47.88]
35-44	47.47 (7.27) [45.23 to 49.71]
45-75	47.29 (8.07) [42.63 to 51.95]
Race	
White	47.14 (7.41) [45.32 to 48.96]
Mixed or multiple	44.27 (5.18) [40.79 to 47.75]
Asian, PI, AI, or AN	45.58 (9.36) [39.63 to 51.53]
Black	48.00 (6.36) [40.10 to 55.90]
Ethnicity	
Hispanic	45.35 (8.09) [43.00 to 47.70]
Non-Hispanic	48.00 (6.36) [46.11 to 49.89]
Language spoken at home	
English	48.50 (6.29) [46.71 to 50.29]
Spanish	43.00 (8.93) [34.74 to 51.26]
Level of education	
High School or less	45.35 (7.08) [42.04 to 48.66]
Some college or technical school	46.80 (7.54) [44.42 to 49.18]
Bachelor’s degree	46.81 (7.29) [43.49 to 50.13]
Professional degree	48.00 (8.06) [42.88 to 53.12]
Insurance type	
Private	46.91 (8.22) [44.47 to 49.35]
Medicaid	46.40 (6.54) [44.50 to 48.30]
Marital status	
Married	47.07 (7.42) [45.27 to 48.87]
Unmarried^a^	44.56 (7.83) [40.39 to 48.73]
Spearman correlation, *r* (95% CI)	
Health literacy	0.30 (0.11 to 0.47)
Acculturation	0.30 (0.02 to 0.54)
Satisfaction with informed consent	0.39 (0.21 to 0.54)
Decisional regret	−0.54 (−0.67 to −0.38)
Socioeconomic status	0.09 (−0.11 to 0.28)

Abbreviations: AI, American Indian; AN, Alaska Native; PI, Pacific Islander.

^a^ Unmarried includes single, divorced, widowed, and separated.

**Table 3.** Multivariable Analysis for Perception of Voluntariness Associated with Language, Health Literacy, Acculturation, Satisfaction With Informed Consent, and Decisional Regret^a^

Variable	Estimate, β (SE) [95% CI]^b^
Spanish language	3.42 (6.49) [−9.30 to 16.15]
Health literacy	4.06 (1.26) [1.60 to 6.53]
Acculturation	4.42 (2.94) [−1.35 to 10.19]
Satisfaction with informed consent	8.15 (5.98) [−3.57 to 19.87]
Decisional regret	2.74 (2.80) [−2.76 to 8.24]

^a^ Risk factors with *P* < .10 from univariable models were included.

^b^ Coefficient estimate, from generalized estimating equations.

### Health Literacy and Sociodemographic Characteristics

In univariable analysis, lower HL was significantly associated with Hispanic ethnicity (mean, 4.16; 95% CI, 3.75-4.57; *P* < .001), Spanish as primary language spoken at home (mean, 3.17; 95% CI, 1.94-4.40; *P* < .001), high school or less educational level (mean, 3.41; 95% CI, 2.83-3.99; *P* < .001), Medicaid insurance type (mean, 4.00; 95% CI, 3.55-4.45; *P* < .001), and single marital status (mean, 3.71; 95% CI, 2.91-4.51; *P* = .03). Parental age and race were not significantly associated with HL (Table 4).

**Table 4.** Univariable Analysis for Health Literacy and Associated Sociodemographic Characteristics (N = 97)

Characteristic	Mean (SD) [95% CI]	*P* value
Age range, y		
18-34	4.46 (1.32) [4.02-4.90]	.41
35-44	4.70 (1.42) [4.25-5.15]	
45-75	4.83 (1.64) [3.79-5.87]	
Race		
White	4.70 (1.30) [4.37-5.03]	.31
Mixed or multiple	4.18 (1.72) [3.02-5.34]	
Asian, PI, AI, or AN	4.90 (1.37) [3.92-5.88]	
Black	4.00 (2.00) [1.52-6.48]	
Ethnicity		
Hispanic	4.16 (1.35) [3.75-4.57]	<.001
Non-Hispanic	5.09 (1.31) [4.69-5.49]	
Language spoken at home		
English	5.20 (1.19) [4.86-5.54]	<.001
Spanish	3.17 (1.17) [1.94-4.40]	
Level of education		
High school or less	3.41 (1.12) [2.83-3.99]	<.001
Some college or technical school	4.58 (1.43) [4.12-5.04]	
Bachelor’s degree	5.14 (1.15) [4.62-5.66]	
Professional degree	5.64 (0.67) [5.19-6.09]	
Insurance type		
Private	5.20 (1.09) [4.88-5.52]	<.001
Medicaid	4.00 (1.45) [3.55-4.45]	
Marital status		
Married	4.77 (1.36) [4.43-5.11]	.03
Unmarried^a^	3.71 (1.38) [2.91-4.51]	

Abbreviations: AI, American Indian; AN, Alaska Native; PI, Pacific Islander.

^a^ Unmarried includes single, divorced, widowed, and separated.

### Secondary Analysis

In univariable analysis, greater decisional regret was significantly associated with lower perception of voluntariness (*r* = −0.54; 95% CI, −0.67 to −0.38; *P* < .001), lower HL (*r* = −0.21; 95% CI, −0.38 to −0.01; *P* = .05), and lower satisfaction with informed consent (*r* = −0.39; 95% CI, −0.54 to −0.21; *P* < .001) (Table 5). There were no significant associations between decisional regret or satisfaction with informed consent and acculturation levels or any of the sociodemographic characteristics. Across all subgroups, mean decisional regret scores were overall low (*x̅* = 20.87; maximum score 100), and mean scores for satisfaction with informed consent were moderately high (*x̅* = 17.44; maximum score 21).

**Table 5.** Univariable Analysis for Decisional Regret, Contextual Factors, and Sociodemographic Characteristics

Variable	Spearman correlation, *r* (95% CI)
Health literacy	−0.21 (−0.38 to −0.01)
Acculturation	−0.22 (−0.47 to 0.06)
Satisfaction with informed consent	−0.39 (−0.54 to −0.21)
Perception of voluntariness	−0.54 (−0.67 to −0.38)
Socioeconomic status	−0.11 (−0.30 to 0.09)

## Discussion

We found that lower perception of voluntariness was significantly associated with lower HL in the multivariable analysis, after adjusting for contextual factors and sociodemographic characteristics. This finding suggests that HL may play a role in perception of voluntary decisions during informed consent. Low HL is associated with poor health outcomes, having an uninsured child, higher hospitalization rates, and decreased access to primary care.^11,12,31,32,33^ Given these challenges, HL is an important skill for successfully navigating the complexities of pediatric cancer treatment that often entails participation in clinical trials. Hispanic individuals with limited English proficiency and low socioeconomic status may have difficulty making informed treatment decisions for their children owing to low HL.^11,33^

Evidence from qualitative studies among Mexican American individuals has shown decisional regret, decreased satisfaction, misperception, and fear associated with clinical trials.^34^ Disparities in clinical trial participation persist among racial/ethnic minority groups, particularly among Hispanic populations, in which participation rates remain very low (0.4%-2.2%).^35^ Improving informed consent for minority groups may lessen misperceptions and facilitate their recruitment into clinical trials.

There is a lack of research assessing perception of voluntariness among Spanish-speaking participants.^26,36^ We found that 10% of participants consented to the leukemia clinical trial in English, but when enrolled in our study, they either answered the English questionnaires in Spanish or requested Spanish language consent forms and questionnaires. This may indicate that these participants did not adequately understand the English informed consent documents for the clinical trial. In addition, parents may feel pressured to enroll their child in a clinical trial because they fear their child’s care will be compromised if they decline participation, particularly when the physician is also the investigator.^37^ Researchers and clinicians can verify comprehension of the informed consent document by rechecking parental understanding through approaches such as open-ended questions and the teach-back method.^38^ If parents are not proficient in English, an experienced professional in-person interpreter is recommended.^39^

Prior studies have reported that lower perceived voluntariness is associated with lower educational and racial/ethnic minority status^26^ and that individuals from racial/ethnic minority groups are less likely to understand voluntariness.^40^ Our results did not show an independent association between Hispanic ethnicity and lower perception of voluntariness. Similarly, Spanish language and low acculturation in Hispanic participants were significantly associated with lower perception of voluntariness in univariable analyses, but these associations did not remain in the multivariable analysis, likely owing to dependence between Spanish language or acculturation and low HL. It is plausible that our study was insufficiently powered to detect an independent association between Hispanic ethnicity and perception of voluntariness or, most likely, that HL may have a stronger association than sociodemographic characteristics, as shown in the multivariable analysis. This finding supports the need for identification of individuals with low HL and the potential role of health literate interventions to improve voluntariness during informed consent for research, focused on concordant language, simplified medical jargon, and discussion of alternative treatments.^5,9,11^

Limited English proficiency and low acculturation have been associated with both low HL and worse health outcomes in the literature.^41,42,43^ Individuals with limited English proficiency and low acculturation are less adapted to the Anglo culture and the English language^44^ and therefore may face HL, language, and cultural challenges when communicating with non-Hispanic clinicians or with clinicians who are not fluent in Spanish. During clinical trial recruitment, it is important to be aware of cultural norms and beliefs of potential participants. Hispanic participants may be more likely to give acquiescent responses that are associated with the Hispanic cultural value of *simpatia*,^45^ which means politeness, accord, and pleasantness in the face of stress.^46^ *Respeto* (respect) is another Hispanic cultural norm that may prevent individuals from asking questions of physicians who are seen as authority figures.^47^ The present study used linguistically and culturally concordant research staff who were fluent in Spanish and familiar with Hispanic cultural values, and few participants declined or withdrew from the study. Hispanic individuals make up the largest racial/ethnic minority group in the US (18.3%).^48^ As this population grows, research teams need to adapt to the changing demographic characteristics and tailor recruitment of Hispanic individuals with low HL into clinical trials. The inclusion of appropriately trained bilingual and bicultural research staff will likely increase recruitment of Hispanic and Spanish-speaking individuals into clinical trials. When delivering informed consent for parents with limited English proficiency and of Hispanic ethnicity, it is important for clinician-investigators to recognize low HL and cultural values that may hinder decision-making and to adapt their informed consent delivery accordingly.

Although decisional regret levels across all subgroups in the sample were relatively low, we found an association between lower HL and greater decisional regret. This may indicate that parents with low HL may feel more regret about their decision to enroll their child in a clinical trial than those with adequate HL. Although participants with lower HL reported lower voluntariness, they remained moderately satisfied with informed consent. Prior literature has found that parents may report satisfaction with the informed consent process even when they do not fully understand the informed consent document.^49,50^ Even parents with limited English proficiency who have reported communication barriers with the medical team have reported high satisfaction with their child’s cancer care.^51^ The disparity that we found between satisfaction and perception of voluntariness during informed consent may reflect parents’ feelings of powerlessness in the face of their child’s illness and the requirement that they make a difficult decision. These individuals may view the overall informed consent process as a separate matter and register the experience in positive terms, highlighting the need to improve understanding of the parents’ role as decision-makers during informed consent.

It has been shown that parents of children with cancer face many obstacles in making decisions, including negative emotions surrounding the diagnosis, uncertainty about their decision, inadequate understanding, time constraints, and ineffective communication with the physician.^52,53,54^ Moreover, clinicians are often faced with the conflict of needing to initiate treatment urgently after the leukemia diagnosis vs giving parents optimal time for informed consent.^14^ Angiolillo et al^55^ reported a staged informed consent across 28 days and showed that this process may decrease anxiety and improve parental understanding of the information presented. In addition to the staged consent process, tailored interventions to improve informed consent, including focus on voluntariness, are needed. Reported interventions include improved communication between parents and physicians during informed consent,^56,57^ parent advocates,^58^ medical interpreters,^39,58^ multimedia video presentations about clinical trials,^59^ and nurse-driven anticipatory guidance.^60^ Given that our results showed that individuals who speak Spanish, are of Hispanic ethnicity, have lower educational levels, and have Medicaid insurance were associated with lower HL, these groups may benefit most from tailored informed consent interventions.

### Limitations

Our study included a high percentage of Hispanic and Spanish-speaking individuals; however, our overall sample was relatively small, included more mothers than fathers, and had a small proportion of Asian/Pacific Islander and Black individuals. Hispanic participants were primarily from Mexico, and although Hispanic individuals of Mexican descent are the largest US Hispanic group (62%),^61^ this may limit the generalizability of our findings to other Hispanic subgroups (eg, individuals from Puerto Rico or from Central or South America). Although we assessed voluntariness soon after the consent decision to mitigate confounding factors, we did not report comprehension of informed consent. We performed only quantitative measurements, limiting the depth of understanding of the complexity of informed consent, including “therapeutic misconception,”^7^ and associated contextual factors, such as patient-clinician communication, and other potentially influencing stressors. In addition, the significant association between HL and voluntariness could be alternatively explained by external influences between the health care system and individuals with low HL, in which imbalanced power dynamics may lead to perceptions that their decisions are “inappropriately influenced by others,” even in health care encounters that do not involve informed consent for research. Moreover, we did not assess outcomes by leukemia types, we did not survey those who declined to participate in the therapeutic clinical trial, and we had a small proportion of participants withdraw from our study; therefore, we were unable to compare outcomes among subgroups, which may limit the generalizability of our results. Finally, we used the NVS instrument^17^ to assess HL because of our past experience with this HL assessment.^62,63^ The strengths of the NVS include that it may be administered in 3 minutes, has been validated in both English and Spanish, and is highly sensitive for detecting limited HL.^17,22^ The NVS primarily tests listening, reading, and numeracy skills, which are key skills in cancer; however, it may not fully address verbal and reasoning skills, writing, and understanding of health terminology. Furthermore, the NVS has not been validated in pediatric oncology settings. Additional studies designed to compare the performance of the NVS with the Short Test of Functional Health Literacy in Adults,^64^ considered by some researchers as the criterion standard instrument to assess HL in research settings, may help with the validation of the NVS as a rapid and valid assessment of HL in the pediatric oncology setting. Future studies should include a more diverse racial/ethnic minority group of participants, parents that have declined clinical trial participation, and characterization of cultural and linguistic concordance among clinicians and parents during informed consent.

## Conclusions

Informed consent requires that study participation is completely voluntary and that all elements of informed consent are understood by the research participant. Although voluntariness is conceptually complex, the significant associations found in the present study between low HL and low perception of voluntariness highlight the need to identify individuals with low HL in a timely manner during clinical trial recruitment and suggest the potential role of culturally, linguistically, and HL-appropriate interventions for underserved individuals to improve voluntary consent. Future research should include qualitative studies to further explore contextual understanding of our results, objective assessments of understanding and retention of the consent document, and evaluation of the role of training in cultural and HL competency for clinicians in enhancing voluntary informed consent. Effective informed consent tailored to the individual’s HL level may contribute to a reduction of disparities in clinical trial participation and equitable translation of discoveries and treatments to underserved groups.
